# Variation in surgical treatment by body mass index in patients with invasive lobular carcinoma of the breast

**DOI:** 10.1007/s10549-024-07452-1

**Published:** 2024-08-11

**Authors:** Israel O. Falade, Kayla M. Switalla, Molly E. Baxter, Astrid Quirarte, Helena Record, Harriet T. Rothschild, Elle N. Clelland, Rita A. Mukhtar

**Affiliations:** 1 School of Medicine, University of California - San Francisco, San Francisco, CA USA; 2 Medical School, University of Minnesota - Twin Cities, Minneapolis, MN USA; 3 Department of Surgery, University of California - San Francisco, San Francisco, CA USA

**Keywords:** Surgery, Lobular, Breast cancer, BMI

## Abstract

**Purpose:**

Patients with invasive lobular carcinoma (ILC) face high rates of positive margins and completion mastectomy, which can be improved with the use of specific techniques, such as oncoplastic surgery. However, prior studies have shown that type of breast cancer surgery performed is also associated with patient factors such as elevated body mass index (BMI). Thus, this study investigates whether BMI impacts the type of surgical interventions in patients with ILC.

**Methods:**

A retrospective analysis of 705 patients with stage I–III ILC from an institutional database was conducted. Patients were stratified by BMI (underweight, normal weight, overweight, obese). Pearson’s Chi-square, ANOVA, and multivariable logistic regression were used to evaluate the relationship between BMI and surgical procedures.

**Results:**

Breast-conserving surgery (BCS) was the initial operation in 60% of patients, with no significant difference by BMI. Among those undergoing BCS, patients with obese BMI were significantly more likely to undergo oncoplastic surgery (46.9% vs. 7.7%, 37.3%, and 33.6% for underweight, normal, and overweight, respectively, *p* = 0.032). Obese BMI patients undergoing mastectomy were less likely to have reconstruction compared to those with underweight, normal weight, and overweight BMI (44.2% vs. 50%, 71.1%, and 64.1%, *p* = 0.002).

**Conclusion:**

Overweight/obese BMI patients with ILC underwent different surgical interventions compared to those with lower BMI. While initial BCS rates were similar, overweight/obese patients had higher oncoplastic surgery rates in BCS and lower reconstruction rates in mastectomy. Further research is needed to understand BMI’s impact on surgical decisions and outcomes in ILC.

## Introduction

Invasive lobular carcinoma (ILC) is the second most prevalent histologic subtype of breast cancer, representing approximately 10–15% of all breast cancers [1]. ILC has distinct biologic and clinical properties that set it apart from the more common invasive carcinoma of no special type [2]. The majority of ILC tumors are strongly estrogen receptor (ER) positive and nearly all lack the adhesion protein E-cadherin, leading to a diffuse growth pattern that is often difficult to detect clinically [3]. Consequently, ILC often presents at more advanced stages. The combination of larger tumors and decreased sensitivity of standard imaging tools presents unique challenges in the surgical management of this disease [4]. Many authors have demonstrated that patients with ILC have higher positive margin rates, higher rates of re-excision, and lower rates of successful breast conservation compared to other subtypes of breast cancer [5–8]. Certain surgical techniques have been shown to reduce positive margin rates, including the use of shave margins and the incorporation of oncoplastic techniques for patients undergoing breast-conserving surgery (BCS). We previously showed this to be true in a cohort of patients with ILC specifically, with a nearly 60% reduction in the odds of positive margins for patients who had shave margins and/or oncoplastic surgery compared to those who had lumpectomy alone [7].

However, investigators have also shown that these additional techniques may lead to increased operative times and possibly increased complication rates compared to lumpectomy alone [9]. This may be especially true for patients with elevated body mass index (BMI). Indeed, BMI has been shown to be associated with surgical outcomes and surgical choice in patients with breast cancer in general, but it has not been studied in ILC. Given the unique aspects of surgical management in ILC, including its diffuse growth pattern and high risk of positive margins, we wondered whether BMI would be associated with the use of shave margins and oncoplastic approaches in ILC. Our primary aim was to determine whether surgical procedures differed by BMI class in a single-institution cohort of patients with ILC of the breast; secondarily, we compared positive margin rates by BMI class.

## Methods

### Study design and data collection

We retrospectively analyzed a prospectively maintained institutional database of patients undergoing surgical treatment for ILC between years 1995 and 2023. We collected data on clinicopathologic features, BMI, type of surgical operation, use of oncoplastic surgery, use of shave margins, and rate of positive surgical margins. Patients who underwent lumpectomy alone, lumpectomy with oncoplastic closure, or oncoplastic reduction mammoplasty (ORM) as their first surgical procedure were considered to have undergone breast-conserving surgery (BCS). For patients who underwent mastectomy, procedures were defined as simple mastectomy, mastectomy with aesthetic flat closure, and mastectomy with reconstruction (either skin-sparing [SSM) or total skin-sparing [TSSM]). Mastectomy with aesthetic flat closure was defined as shaping and smoothing soft tissue to create a flat chest contour, often involving de-epithelizing skin flaps, removal of extra subcutaneous fat, and obliteration of the inframammary fold, with most cases at our institution being performed by a plastic surgeon concurrent with simple mastectomy [10, 11]. For those undergoing BCS, we estimated lumpectomy specimen volume in cm^3^ by multiplying lumpectomy specimen diameter in three dimensions, are recorded from pathology reports. We recorded the use of shave margins after both BCS and mastectomy procedures. Margins were considered positive if pathologic evaluation of final margins indicated “ink on tumor.” We excluded patients who were found to have de novo stage IV disease and those missing a recorded BMI at the time of surgery. BMI groups were defined according to the World Health Organization classifications: underweight (BMI < 18.5 kg/m^2^); normal weight (BMI 18.5–24.9 kg/m^2^); overweight (BMI 25–29.9 kg/m^2^); and obese (BMI ≥ 30 kg/m^2^).

### Statistical analysis

Patient and tumor characteristics were compared across BMI classes using ANOVA. Chi-squared tests were used to analyze the associations between BMI class and type of surgical operation, use of oncoplastic surgery, use of shave margins, and rate of positive surgical margins. Logistic regression models were developed to evaluate the relationship between BMI category and other variables. All analyses were performed using STATA 16.1 (Stata Corp., College Station, TX, USA) with two-tailed *p* values < 0.05 indicative of statistical significance.

## Results

### Patient and tumor characteristics overall and by BMI

We identified 713 patients with stage I–III ILC in our institutional database who had available BMI data. The average age at diagnosis was 59.8 years (ranging from 21.1 to 91.2) and the majority of patients identified as white (78.4%). Average tumor size was 3.2 cm (standard deviation 3.0), and 69.9% patients were pathologically node negative with the remaining being node positive. Tumor receptor subtype was ER positive, PR positive, and HER2 negative in the majority of cases (72.5%). Tumor grade was 2 in the majority (67.2%) of patients, and 40 tumors (5.8%) had lymphovascular invasion (Table 1).

**Table 1:** Demographic and clinicopathologic features in patients with ILC stratified by weight class

	Total	Underweight	Normal weight	Overweight	Obese	*p* value
	*n* = 713	*n* = 21	*n* = 356	*n* = 199	*n* = 137	
Age at diagnosis	59.8 (12)	58.1 (13.1)	58.3 (11.7)	60.1 (12.3)	63.6 (11.6)	< 0.001
ILC tumor diameter (cm)	3.2 (3.0)	3.4 (3.5)	2.9 (2.8)	3.3 (3.0)	3.7 (3.1)	0.02
Positive nodes	1.3 (3.9)	0.3 (1.3)	1.1 (3.4)	1.6 (4.6)	1.6 (4.2)	0.27
ILC receptor subtype						0.11
ER+PR+HER−	492 (72.5%)	11 (52.4%)	239 (70.3%)	135 (73.0%)	107 (80.5%)	
ER+PR−HER−	124 (18.3%)	5 (23.8%)	63 (18.5%)	36 (19.5%)	20 (15.0%)	
ER−PR−HER−	17 (2.5%)	1 (4.8%)	11 (3.2%)	3 (1.6%)	2 (1.5%)	
HER2+	46 (6.8%)	4 (19.1%)	27 (7.9%)	11 (6.0%)	4 (3.0%)	
ILC grade						0.38
1	188 (27.2%)	6 (28.6%)	85 (24.6%)	61 (31.9%)	36 (26.8%)	
2	465 (67.2%)	13 (61.9%)	243 (70.2%)	122 (63.9%)	87 (64.9%)	
3	39 (5.6%)	2 (9.5%)	18 (5.2%)	8 (4.2%)	11 (8.2%)	
LVI	40 (5.8%)	0	17 (4.9%)	13 (7.0%)	10 (7.6%)	0.38

Data are presented as mean (SD) for continuous measures and *n* (row %) for categorical measures and *n* (column %) for binary measures

Not all patients had complete demographic information, leading to varying sample sizes for certain variables

*ILC* invasive lobular carcinoma, *ER* estrogen receptor, *PR* progesterone receptor, *HER2*: human epidermal growth factor receptor 2, *LVI* lymphovascular invasion

When stratified by BMI class, 21 patients (2.9%) were classified as underweight, 356 patients (50%) as normal weight, 199 patients (28%) as overweight, and 137 patients (19%) as obese. Obesity was associated with older age at diagnosis and larger tumor size. In those with overweight/obese class BMI, average age at diagnosis was 61.5 years, compared to 58.3 years in those with underweight/normal class BMI (*p* = 0.0002). Average tumor size was 2.9 cm in underweight/normal weight cases versus 3.5 cm in those with overweight/obese class BMI (*p* = 0.009). There was no association between BMI class and number of positive nodes, tumor grade, tumor receptor subtype, or presence of lymphovascular invasion (Table 2).

**Table 2:** Procedure and clinicopathologic outcomes of patients with ILC stratified by weight class

	Total	Underweight	Normal weight	Overweight	Obese	*p* value
	*n* = 713	*n* = 21	*n* = 356	*n* = 199	*n* = 137	
First procedure						1.0
BCS	425 (60.3%)	13 (62.0%)	212 (60.0%)	119 (60.4%)	81 (61.0%)	
Mastectomy	280 (39.7%)	8 (38.1%)	142 (40.1%)	78 (39.6%)	52 (39.1%)	
BCS type						0.007
Lumpectomy	267 (62.8%)	12 (92.3%)	133 (62.7%)	79 (66.4%)	43 (53.1%)	
Lumpectomy with OC	79 (18.6%)	1 (7.7%)	46 (21.7%)	20 (16.8%)	12 (14.8%)	
ORM	79 (18.6%)	0	33 (15.6%)	20 (16.8%)	26 (32.1%)	
Mastectomy type						0.002
Mastectomy	10 (3.6%)	1 (12.5%)	5 (3.5%)	3 (3.9%)	1 (1.9%)	
Mastectomy with AC	92 (32.9%)	3 (37.5%)	36 (25.4%)	25 (32.1%)	28 (53.8%)	
SSM	41 (14.6%)	1 (12.5%)	15 (10.6%)	15 (19.2%)	10 (19.2%)	
TSSM	137 (48.9%)	3 (37.5%)	86 (60.6%)	35 (44.9%)	13 (25.0%)	
Immediate reconstruction following mastectomy	178 (63.6%)	4 (50%)	101 (71.1%)	50 (64.1%)	23 (44.2%)	0.006
Shave margins used						0.02
BCS	253 (63.3%)	5 (38.5%)	128 (63.4%)	65 (58.0%)	55 (75.3%)	0.03
Mastectomy	87 (41.8%)	0	49 (43.8%)	22 (40.7%)	16 (43.2%)	0.28
Positive margins						
BCS	151 (36.0%)	3 (23.1%)	78 (37.1%)	41 (35.0%)	29 (36.7%)	0.77
Mastectomy	33 (12.3%)	1 (14.3%)	17 (12.3%)	9 (12.3%)	6 (12.0%)	1.0
Radiation						
BCS	253 (60.7%)	7 (58.3%)	121 (57.9%)	71 (60.9%)	54 (68.4%)	0.45
Mastectomy	90 (32.7%)	3 (3.33%)	38 (42.2%)	33 (36.7%)	16 (32.0%)	0.13
Mean lumpectomy volume, cm^3^ T1 T2 T3	93.8 69.5 102.3 194.4	33.9 26.7 50.6 27.1	66.5 49.8 83.9 124.7	97.3 77.5 98.4 276.8	164.6 143.7 144.8 237.6	< 0.001 < 0.001 0.02 0.11
Second surgery type^a^						0.84
Re-excision	100 (61.7%)	2 (50%)	55 (64.7%)	26 (57.8%)	17 (60.7%)	
Completion mastectomy	62 (38.3%)	2 (50%)	30 (35.3%)	19 (42.2%)	11 (39.3%)	

Data are presented as mean (SD) for continuous measures and *n* (row %) for categorical measures and *n* (column %) for binary measures

Not all patients had complete demographic information, leading to varying sample sizes for certain variables

*BCS* breast-conserving surgery, *OC* oncoplastic closure, *AC* aesthetic flat closure

^a^Among patients who underwent BCS and had second operation

### Surgical procedures and BMI

A total of 705 patients underwent surgical treatment, and 8 patients omitted surgical intervention for unknown reasons. Overall, breast-conserving surgery (BCS) was the initial operation in 60% of patients and mastectomy in 40%. This did not differ by BMI, with BCS performed in 62.0, 60.0, 60.4, and 61.0% of patients in the underweight, normal weight, overweight, and obese BMI groups, respectively.

Among all patients undergoing BCS (*n* = 425), 267 (62.8%) had lumpectomy alone, 79 (18.6%) had lumpectomy with oncoplastic closure, and 79 (18.6%) had ORM. This differed significantly by BMI class, as those with obese BMI were significantly more likely to have an oncoplastic surgical approach (either lumpectomy with oncoplastic closure or ORM) compared to those with underweight, normal weight, and overweight BMI (46.9% versus 7.7%, 37.3%, and 33.6%, respectively, *p* = 0.032). Average lumpectomy specimen volume was 93.9 cm^3^ overall and was significantly smaller in those with lower BMI categories compared to higher BMI categories (mean volume 33.9 cm^3^, 66.5 cm^3^, 97.4 cm^3^, and 164.6 cm^3^ in underweight, normal weight, overweight, and obese BMI groups, respectively, *p* < 0.0001). This relationship was true for patients with T1 and T2 tumors, but among those with T3 tumors there was no difference in mean lumpectomy volume by BMI group.

Among all patients undergoing mastectomy (*n* = 280), 10 (3.6%) had simple mastectomy, 92 (32.9%) had mastectomy with aesthetic flat closure, and 178 (63.6%) had mastectomy with reconstruction (SSM or TSSM). This varied significantly by BMI class, with higher rates of aesthetic flat closure and lower rates of TSSM in patients with obese BMI (*p* = 0.002). Rates of aesthetic flat closure for underweight, normal weight, overweight, and obese BMI groups were 37.5, 25.4, 32.1 and 53.8%, respectively. Rates of TSSM for underweight, normal weight, overweight, and obese BMI groups were 37.5, 60.6, 44.9, and 25%, respectively. Immediate reconstruction following mastectomy was least common among those with obese BMI compared to underweight, normal weight, and overweight BMI (44.2% versus 50%, 71.1%, and 64.1%, respectively, *p* = 0.006).

### Use of shave margins

Overall, shave margins were utilized in 340 patients (55.9%) and more frequently among patients having BCS compared to mastectomy (63.3% vs 41.8%, respectively). Overall, the use of shave margins varied significantly by BMI group (27.8, 56.4, 52.4, and 64.6% in underweight, normal weight, overweight, and obese BMI groups, respectively, *p* = 0.02). Among patients having BCS, shave margin use was significantly less common in those with underweight range BMI, and more common in those with obese range BMI (38.5, 63.4, 58.0 and 75.3% in underweight, normal weight, overweight, and obese BMI groups, respectively, *p* = 0.025). Among patients having mastectomy, shave margin use was not associated with BMI class.

### Positive margin rates

Among the 687 cases with available margin data, 26.8% of patients had initial positive margins. For those undergoing BCS, 36.0% had initial positive margins, while for those undergoing mastectomy, 12.3% had initial positive margins. These rates did not differ by BMI.

Among patients undergoing BCS, the use of shave margins was strongly associated with lower rates of positive margins (29.8% versus 48.3%, *p* < 0.001). Importantly, the association between shave margins and lower positive margin rates was seen both among patients with underweight/normal category BMI and among patients with overweight/obese category BMI. In the underweight/normal category BMI group who underwent BCS, positive margins rates were 29.5% versus 46.3% in those with and without shave margins, respectively (*p* = 0.013). In the overweight/obese BMI group who underwent BCS, positive margin rates were 25.8% versus 47.7% in those with and without shave margins, respectively (*p* = 0.003).

In a multivariate logistic regression model adjusting for tumor size, the use of oncoplastic surgery was associated with a significant reduction in the odds of positive margins for patients with overweight/obese group BMI who underwent BCS (odds ratio [OR] 0.42, 95% confidence interval 0.21–0.85, *p* = 0.015). In those with underweight or normal group BMI who underwent BCS, oncoplastic surgery was not significantly associated with reduced odds of positive margins (OR 0.68, 95% CI 0.36–1.3, *p* = 0.231).

## Discussion

In this analysis, we found that surgical management of stage I–III ILC of the breast varied significantly by BMI. While rates of mastectomy overall were similar across BMI groups, the use of oncoplastic approaches, immediate reconstruction, and shave margins differed significantly. Although several series have reported on differences in the surgical approach to breast cancer based on BMI, none have focused specifically on patients with ILC [12–14]. Because of its diffuse growth pattern, ILC has distinct surgical challenges, including discordance between tumor size on imaging versus on final pathology, increased rates of positive margins, and increased need for mastectomy compared to patients with the more common IDC or carcinoma of no special type. [5, 15–17]

The literature shows an inconsistent relationship between BMI and utilization of various breast surgery procedures. While we found no difference in rates of attempted BCS by BMI group, other investigators have shown that patients with overweight/obese BMI are more likely to undergo BCS than mastectomy [13, 14]. This could be related to a preference to avoid longer anesthesia times in the setting of co-morbid conditions associated with elevated BMI, but could also reflect a preference for BCS if reconstruction after mastectomy is less likely to be offered [18, 19]. A limitation of our study is lack of information on which surgical procedures were recommended by surgeons and reasons for selecting procedures by patients.

In our study population, the lack of higher rates of BCS in those with higher BMI could reflect institutional practices, but could also be specific to those with ILC. Because ILC grows in a diffuse pattern and presents at higher stages, mastectomy is more common in this tumor type compared to the more common carcinoma of no special type. Interestingly, Tong et al*.* showed that rates of surgical complications after ORM were lower than after mastectomy with reconstruction in those with obese category BMI [20]. These findings suggest that utilization of ORM may allow for avoidance of mastectomy with fewer complications. For patients with ILC, the ability to safely offer ORM can improve outcomes, as positive margin rates are reduced; this was also observed in this analysis across BMI groups.

We and others previously showed that both shave margins and oncoplastic surgery are associated with significantly reduced risk of positive margins specifically for those with ILC [5, 21, 22]. In this analysis stratified by BMI, we found that shave margins were associated with lower incidence of positive margins regardless of BMI. Interestingly, oncoplastic surgery was only associated with lower odds of positive margins for those with overweight or obese BMI. In this group, the use of oncoplastic surgery at the time of lumpectomy resulted in a 58% reduction in the odds of positive margins when adjusting for tumor size. In contrast, for those with underweight or normal weight BMI, the use of oncoplastic surgery at the time of lumpectomy was also associated with lower odds of positive margins, but this did not reach statistical significance.

For those with higher BMI, these findings are reassuring and suggest that shave margins and oncoplastic surgical approaches are helpful tools to reduce positive margin risk. For those with lower BMI, use of both shave margins and oncoplastic approaches were less common. We have not seen this reported in the literature before and hypothesize that oncoplastic approaches may be less common possibly due to smaller breast size and reduced options for approaches, such as reduction mammoplasty. One limitation of this study is that breast size was unavailable as a variable to evaluate. Potentially smaller breast size could also have reduced the likelihood of surgeons taking shave margins, which were associated with significantly reduced positive margins in this group.

These findings suggest the need for further investigation into the drivers of these disparate approaches to the surgical management of ILC. For those with overweight/obese BMI, reduced rates of immediate reconstruction warrant additional research into the risks of complications, particularly compared to lumpectomy with oncoplastic approaches. For those with underweight/normal BMI, more study is needed to understand the lower utilization of shave margins and oncoplastic surgery. It is possible that the lower rates of oncoplastic surgery in this group limited our ability to detect a significant association with positive margin rates.

In this study, we observed that oncoplastic approaches were less frequently used in underweight and normal weight patients, possibly due to smaller breast sizes. Although we did not collect breast size, we did note that mean lumpectomy specimen volume was significantly smaller in those with lower BMI categories, possibly suggesting a relationship between breast size and BMI. During the study period, the oncoplastic approaches utilized were primarily volume displacement techniques, which are limited by smaller breast volume. Recent literature suggests that volume replacement methods can allow for larger resections even in women with smaller breast size, resulting in reduced positive margin rates [23]. These approaches have been incorporated into our current institutional practice.

Additional limitations of this study include the retrospective design and the single-institution population, potentially limiting the generalizability of findings. The choice of surgical procedure was also likely subjected to biases from both patients and physicians and our data does not allow us to determine why certain procedures were chosen or not chosen for patients in different BMI categories. Factors such as comorbidities, insurance coverage, and surgeon experience may also have influenced the choice of surgery. Finally, while the long study period included allows for larger sample size, changes in institutional practice over time may have influenced performance of certain procedures.

## Conclusion

In conclusion, our analysis of 713 patients with stage I–III ILC showed that choice of surgical procedure for ILC is influenced by BMI. We demonstrated that patients with lower BMI were less likely to undergo oncoplastic surgical approaches, while those with higher BMI were more likely to undergo aesthetic flat closure after mastectomy instead of reconstruction. The use of shave margins in BCS was associated with reduced risk of positive margins but was not utilized equally across BMI groups. Our study supports the use of shave margins across patients with all BMI classes and highlights the need for further study to identify factors associated with improved surgical outcomes across the range of BMI.

## Data Availability

No datasets were generated or analysed during the current study. The datasets generated during and/or analyzed during the current study are not publicly available due to patient privacy and HIPAA reasons but are available from the corresponding author on reasonable request.
